# Quantifying Indirect Billing Within the Medicare Physician Fee Schedule

**DOI:** 10.1001/jamahealthforum.2025.0433

**Published:** 2025-04-11

**Authors:** John F. Mulcahy, Sadiq Y. Patel, Ateev Mehrotra, Hannah T. Neprash

**Affiliations:** 1 Division of Health Policy and Management, University of Minnesota School of Public Health, Minneapolis; 2 Clinical Product Development, Waymark, San Francisco, California; 3 School of Social Policy and Practice, University of Pennsylvania, Philadelphia; 4 Department of Health Services, Policy and Practice, Brown University School of Public Health, Providence, Rhode Island

## Abstract

**Importance:**

Under certain circumstances, advance practice clinicians (APCs), such as physician assistants and nurse practitioners, can bill Medicare directly or indirectly (ie, incident to the services of a physician). With indirect billing, the submitted claim states the care was provided by the physician, and the reimbursement is higher.

**Objective:**

To quantify volume and spending on office-based encounters billed indirectly in the Medicare program.

**Design, Setting, and Participants:**

This cohort study used Medicare fee-for-service and Medicare Advantage claims data to identify indirectly billed APC services. To do so, office-based Medicare Part B claims (ie, clinician services) were linked to Part D claims for prescription drug fills. Because the latter contains the prescribing clinician’s unique identifier, this linkage distinguished between directly and indirectly billed services provided by APCs. In this way, the fraction of encounters and component services billed indirectly by APCs and physicians were quantified.

**Main Outcomes and Measures:**

Share of fee-for-service and Medicare Advantage office encounters provided by APCs and billed indirectly. Share of a physician’s billed claims actually provided by an APC and billed indirectly.

**Results:**

In 2022, of all office encounters provided by an APC, 38.9% were billed indirectly. Conversely, for the median physician in 2022, indirect billing on behalf of APCs represented 11.1% of all billed encounters. Billing for care delivered by APCs was most common among surgical specialists (29.7% of encounters) and least common for primary care physicians (3.9%). If all indirectly billed APC-provided care was billed directly by the APC, Medicare would have saved $270 million in 2022.

**Conclusions and Relevance:**

The results of this cohort study suggest that APCs provide a substantial fraction of office-based care received by Medicare beneficiaries. Identifying indirectly billed APC-provided care is integral to understanding who serves Medicare beneficiaries.

## Key Points

**Question:**

How prevalent is indirect billing among advanced practice clinicians (APCs) providing office-based care?

**Findings:**

In this cohort study of clinicians billing Medicare, APCs billed indirectly for 38.9% of all office-based encounters with Medicare beneficiaries in 2022, representing potential annual savings of $270 million to Medicare had indirectly billed services been billed directly. For the median physician, indirect billing on behalf of APCs represented 11.1% of their billed encounters in 2022, with higher rates among surgical specialists and lower rates among primary care physicians.

**Meaning:**

The study results suggest that indirect billing in Medicare is common, underrepresenting the role of APCs while exaggerating the work of physicians.

## Teaser

This cohort study examines volume and spending on office-based encounters billed indirectly by advanced practice clinicians in the Medicare program.

## Introduction

Nurse practitioners (NPs) and physician assistants (PAs) represent a rapidly growing share of the clinical workforce.^1,2^ Researchers have historically struggled to accurately observe the care provided by these and other advanced practice clinicians (APCs) in Medicare administrative data and other sources of health insurance claims data. This challenge is the result of Medicare payment policy that allows APCs to bill Medicare directly (ie, using their own national provider identifier [NPI]) or indirectly, for which services are billed as incident to a physician. The insurance claim resulting from indirectly billed services is not flagged and only includes the physician’s identifier. Directly billed services by an APC are paid at 85% of what is paid to a physician.^3^

Indirect billing began with the 1977 Rural Clinic Act and expanded under the Balanced Budget Act of 1997.^4,5^ Indirect billing is only permitted under specific circumstances, with restrictions on the types of patients, services, and settings in which indirect billing can occur. For instance, indirect billing can only occur for established patients (ie, not new patients) and is not allowed for any Medicare Part A services, nor in certain settings of care (eg, hospital outpatient departments).^6,7^ Despite such restrictions, evidence suggests that indirect billing may not always comply with Medicare payment policy.^8,9,10^

Indirect billing is simultaneously associated with an undercount of services provided by APCs and overcount of services provided by physicians. Accurately identifying care provided by APCs vs physicians may improve Medicare payment policy. For example, clinician-level quality metrics may be more accurate if calculated using the full sample of visits provided by each clinician. Identifying who treated patients rather than who billed for their care may also improve the accuracy of patient attribution for alternative payment models, such as accountable care organizations.

In prior work, we developed a method for identifying indirect billing in Medicare claims data.^10,11^ As detailed later in the article and the eAppendix in Supplement 1, this approach relies on clinician identifiers included on prescription drug claims to identify evaluation and management (E/M) visits likely rendered by an APC (as the prescribing clinician) but billed indirectly (ie, incident to a physician). We found that incident to billing is common among E/M services for established patients in the traditional Medicare program,^10,11^ estimating that more than 40% of all E/M office visits for NPs for established patients are billed indirectly.^6^

This article advances this prior work in 2 ways. First, we examined all indirect billing of office-based services by NPs, PAs, and clinical nurse specialists (CNS). Prior analyses focused only on E/M services. Because indirect billing is permitted for other services within Medicare’s Physician Fee Schedule (PFS), documenting the broader patterns of indirect billing beyond E/M services is essential to fully understanding the role of APCs in serving Medicare beneficiaries and the potential savings to Medicare, were it to disallow indirect billing. Second, we examine whether indirect billing occurs in the Medicare Advantage (MA) program. Private insurers have taken steps to limit indirect billing. For example, for some period UnitedHealth Group prohibited indirect billing but then reversed course, and its policy was consistent with traditional Medicare’s payment policy and allowed indirect billing.^12,13^ Whether these policy shifts were associated with changes in care with MA is unknown.

## Methods

Because only deidentified administrative data were used, this cross-sectional study was deemed exempt from institutional review board review by the institutional review board at the University of Minnesota. This study followed the Strengthening the Reporting of Observational Studies in Epidemiology (STROBE) reporting guideline.

### Study Population

Using 100% of Part B FFS Medicare and 100% of MA claims for services occurring in office settings from 2016 to 2022, we limited our analysis to claims for services billed by physicians and APCs (ie, NPs, PAs, and CNSs). From these claims, we collapsed the analysis to the encounter level, defining an encounter as a combination of beneficiary identifier, service date, and clinician identifier (ie, NPI). Across all encounters, we limited our analyses to those that included at least 1 service on the Medicare PFS that was frequently (ie, representing ≥0.1% of APC direct billing volume) performed and billed directly by APCs (eTable 1 in Supplement 1). Nearly all (97.5%) encounters included an E/M service, and 1 in 3 encounters also included a non–E/M service.

We then searched for and linked Part D claims for prescription drug fills, matching to encounters on beneficiary identifier and service date (plus or minus 1 day, to account for the time it may have taken beneficiaries to fill prescriptions and/or inaccuracies in reported visit date). In linking encounters to prescriptions, we prioritized exact date matches. The few encounters linked to a prescription written by a physician and a different prescription written by an APC on the same date were excluded from our sample. See eFigure 1 in Supplement 1 for a visual depiction of this process and the eAppendix in Supplement 1 for additional details on each step.

Among encounters linked to a prescription, we classified those billed by an APC as directly billed care provided by an APC. For encounters billed by a physician, we assessed whether the linked prescription listed the same physician as the prescribing clinician. If so, the encounter was classified as directly billed and provided by a physician. Indirectly billed encounters were those that were billed by a physician but in which the linked prescription identified an APC as the prescriber.

We repeated this process in MA encounter data. Following convention, we limited our analysis to MA contracts that were previously validated as exhibiting high data completeness (ie, ≤10% missing hospital stays and ≤10% difference in ambulatory and emergency department visits between encounter data and Healthcare Effectiveness Data and Information System data).^14^

For each encounter, we recorded characteristics of the encounter itself, patient, and billing clinician. Encounter characteristics included Berenson-Eggers Type of Service classification. Encounters were allowed to have multiple Berenson-Eggers Type of Service classifications when they comprised multiple services. Patient characteristics included sex, age, self-reported race and ethnicity, rurality, disability status, dual eligibility, chronic condition indicators, and census division of residence. Billing clinician characteristics included credential (physician, NP, PA, and CNS) and specialty (only available for physicians).

### Extrapolating to Encounters Without a Prescription

The previously described methods only identify indirect billing for encounters provided by APCs when those encounters were linked to a prescription drug fill (24.7% of all visits). We extrapolated patterns of indirect billing within the prescription-linked encounters to estimate indirect billing prevalence among encounters without linked prescriptions. In brief, we used all prescription-linked Medicare FFS encounters billed by physicians in an office setting, regressing an indicator for indirect billing (ie, whether that encounter was actually provided by an APC as identified using the previously described methods) on encounter, physician, and patient characteristics. We then applied coefficients (eTable 2 in Supplement 1) to calculate the predicted likelihood of indirect billing for each prescription-unlinked, physician-billed office encounter and an overall expected rate of indirect billing, which we applied to the count of prescription-unlinked encounters to estimate the number of indirectly billed prescription-unlinked encounters in every year. We applied the overall expected rate of indirect billing to total allowed PFS charges to estimate Medicare spending on indirectly billed prescription-unlinked encounters. We repeated this process in the MA sample (for volume only, because spending information was not available), omitting chronic condition indicators as a predictor because these are derived from FFS claims. See the eAppendix in Supplement 1 for additional details on this process.

### Calculating the Physician-Level Share of Misattributed Encounters

For every physician, we calculated their annual share of misattributed encounters, which were defined as the share of prescription-linked visits billed under their NPI but actually provided by APCs. We then plotted the distribution of this physician-level variable, stratifying by broad specialty category. To avoid including physicians for whom Medicare represented a very small share of their patient panel, this analysis included only physicians billing at least 25 visits annually. Sensitivity analyses tested whether a higher threshold (eg, 50 visits) altered findings.

### Calculating Encounter-Level Spending

To quantify the budgetary implications of indirect billing, we summed allowed charges for all services reimbursed under the PFS, as the payment differential for direct vs indirect billing applies only to PFS services. Spending analyses excluded encounters for MA enrollees because MA claims data do not include paid amounts.

## Results

**Figure 1.**
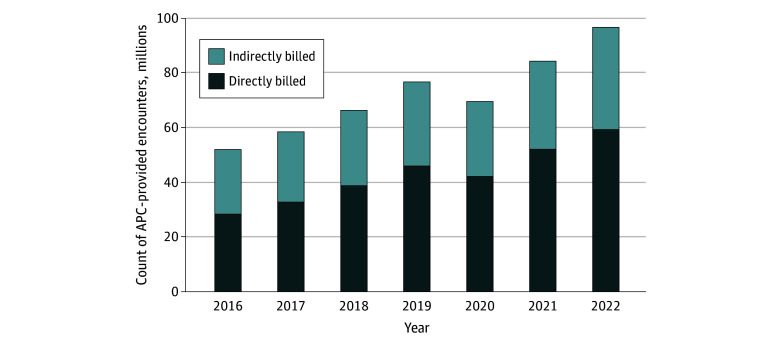
Volume of Advanced Practice Clinician (APC)–Provided Encounters in Medicare Advantage and Fee for Service by Billing Status From 2016 to 2022 Annual volume and share of all APC-provided encounters for Fee for Service and Medicare Advantage by billing status.

**Figure 2.**
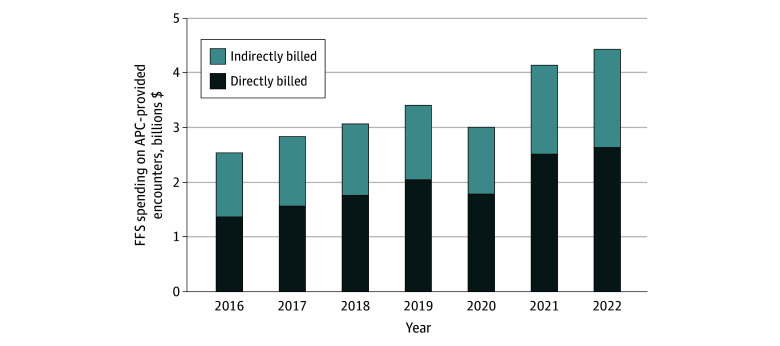
Medicare Fee-for-Service (FFS) Spending of Advanced Practice Clinician (APC)–Provided Encounters by Billing Status From 2016 to 2022 Annual volume and share of all APC-provided FFS physician fee schedule–allowed charges by billing status.

Figure 1 displays the number and share of APC-provided encounters by billing status (ie, direct vs indirect) and over time. In 2022, 37.6 million APC-provided encounters were billed indirectly, representing 38.9% of all APC-provided encounters for Medicare beneficiaries (FFS and MA). In 2022, Medicare FFS spent $4.4 billion on APC-provided PFS care, 41% of which was billed indirectly (Figure 2; eTable 3 in Supplement 1).

Patterns of indirect billing have changed over time (Figure 1; eTable 3 in Supplement 1). As a share of all APC-provided encounters, indirect billing declined from 45.1% in 2016 to 38.9% in 2022. As a share of FFS PFS spending on APC-provided encounters, indirect billing also declined from 45.8% in 2016 to 40.6% in 2022 (Figure 2; eTable 3 in Supplement 1). However, the absolute number of indirectly billed APC-provided encounters (and the spending on those encounters) has increased over time.

Indirect billing is more common in MA vs FFS. In 2022, a lower share of APC-provided visits were billed indirectly in FFS vs MA (35.7% vs 43.7%; eFigure 2 in Supplement 1).

**Figure 3.**
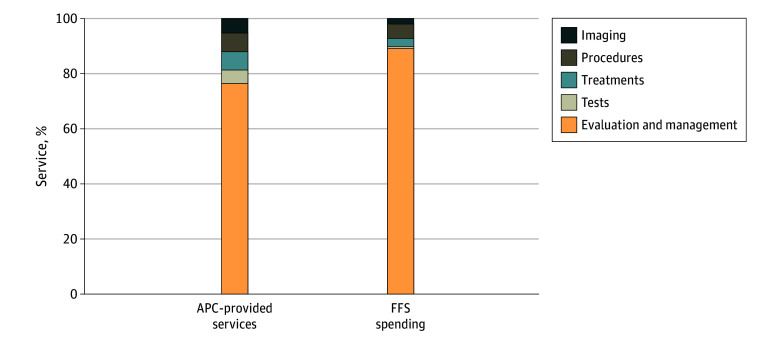
Share of Indirectly Billed Advanced Practice Clinician (APC)–Provided Services and Fee-for-Service (FFS) Spending by Service Category in 2022 Total service count and total spending for APC-provided indirectly billed services provided to FFS Medicare beneficiaries. Only indirectly billed FFS services linked to prescriptions were included.

E/M services account for most indirectly billed services. Across all services provided by APCs and billed indirectly, 76.7% were E/M, 4.5% were tests, 6.7% were treatments, 6.6% were procedures, and 5.4% were imaging services (Figure 3). As a share of total reimbursements for indirectly billed care in the FFS population, E/M represented 89.2%, tests were 0.7%, treatments were 3.0%, procedures were 5.2%, and imaging services were 1.9%. A list of the dozen most frequently indirectly billed services (in descending order of frequency) is available as eTable 4 in Supplement 1.

**Figure 4.**
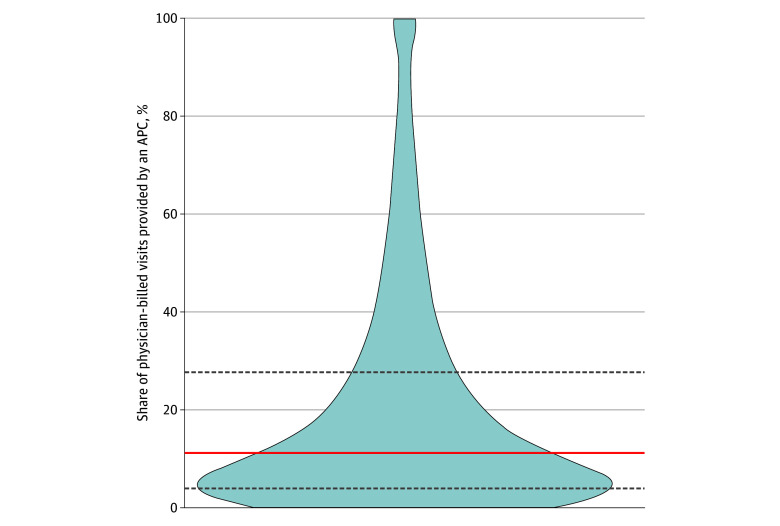
Physician-Level Share of Encounters Provided by Advanced Practice Clinicians (APCs) in 2022 Distribution of physicians by share of prescription-linked visits billed under their national provider identifier and provided by APCs (ie, billed indirectly). Calculations included only prescription-linked visits.

We next analyzed results on the frequency of indirect billing, from the physician perspective. As a share of the total Medicare billing from physicians, indirect billing for APC-provided care has grown substantially over time. Arranging physicians by share of total claims billed on behalf of APCs, the median physician in 2022 billed indirectly for 11.1% of encounters, up from 6.5% of encounters in 2016 (Figure 4; eTable 6 in Supplement 1). Across all office-based encounters billed by physicians, 14% were actually provided by APCs and billed indirectly in 2022, up from 9% in 2016 (eTable 5 in Supplement 1).

**Figure 5.**
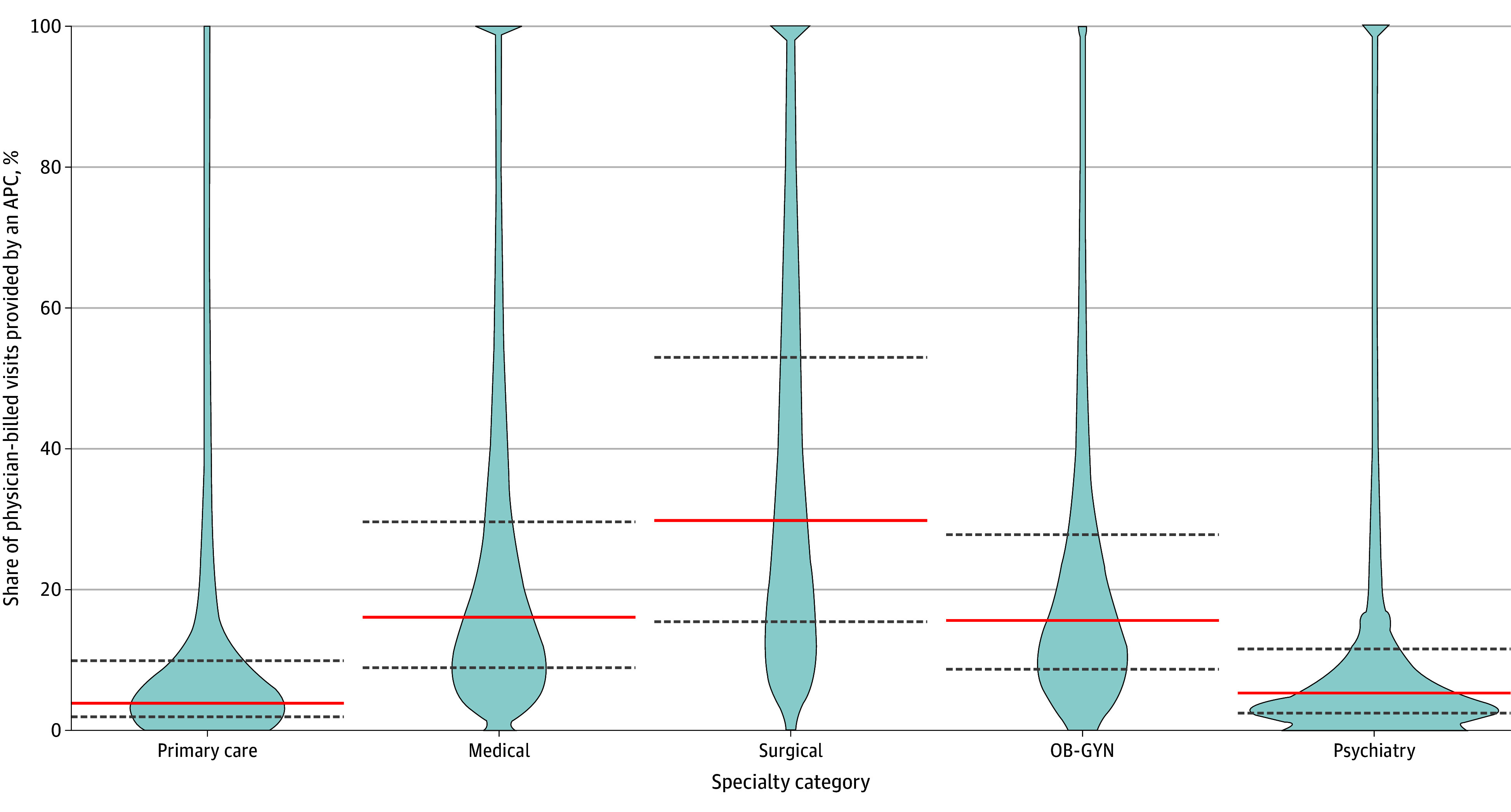
Physician-Level Share of Visits Provided by Advanced Practice Clinicians (APCs) by Specialty Category in 2022 Distribution of physicians by share of prescription-linked visits billed under their national provider identifier and provided by APCs (ie, billed indirectly), stratifying by specialty category. Calculations included only prescription-linked visits. OB-GYN indicates obstetrician-gynecologist.

Use of indirect billing varied by physician specialty, with the highest rates observed among surgical specialists (29.7% in 2022, for the median physician) and the lowest among primary care physicians (3.9%; Figure 5). Increasing the inclusion threshold for physicians from 25 to 50 Medicare visits yielded similar results (eFigure 3 in Supplement 1).

## Discussion

In this cohort study, we estimated that 38.9% (37.6 million) of all APC-provided office encounters were billed indirectly (ie, incident to the services of a physician) in 2022. While the share of APC-provided encounters billed indirectly has decreased over time, the absolute number of indirectly billed visits is increasing. Spending on indirectly billed APC-provided care has also increased nearly every year. In 2022, FFS Medicare expenditures for PFS services delivered during indirectly billed APC-provided encounters totaled $1.8 billion. Had this care been billed directly (and reimbursed at 85% of Medicare’s PFS rates), Medicare would have saved $270 million.

Indirect billing of APC-provided care simultaneously underrepresents the clinical work of APCs while misrepresenting the role of physicians in care delivery. This distortion varies by physician specialty, with surgical specialists billing on behalf of APCs most and primary care physicians least. This finding suggests growing inaccuracies in alternative payment models and value-based care models that rely on the billing clinician to attribute patients to clinicians and/or calculate clinician-level quality measures.

We found that indirect billing rates were higher in MA vs FFS. This was somewhat surprising, given the financial incentive that MA plans have to reduce expenditures and the efforts some insurers have put into reducing indirect billing by APCs. This may simply reflect the outsized effect of Medicare’s PFS in determining payment structures and rates among commercial insurers.^15^ Physicians are unlikely to practice differently among clinically similar patients with MA vs FFS insurance coverage.

Although indirect billing can be used across many services, it is largely used for E/M services. Non–E/M services make up 23% of encounters billed indirectly and 11% of spending on indirectly billed services. Eliminating indirect billing of APC-provided would generate substantial savings, both for the Medicare program and Medicare beneficiaries, whose Part B cost-sharing typically requires that they pay 20% out of pocket. In total, Medicare would save roughly a quarter of a billion dollars annually in FFS spending and presumably similar savings in MA if indirectly billed services were billed directly. While these are substantial amounts in dollars, this represents only a sliver of total Medicare spending in 2022 ($944 billion). These savings estimates also presume that it is appropriate to pay APCs $0.85 on the dollar for the same service provided by a physician. This relative discount is a historical legacy, and there is ongoing debate on whether it should be continued. Shrinking the gap between physician and APC reimbursement would reduce the budgetary savings generated by eliminating indirect billing.

Our findings demonstrate how important it is to identify indirectly billed visits when quantifying the role of APCs in caring for Medicare patients. Failing to do so would undercount APC-provided visits by two-fifths and overestimates the work of physicians. Our methods potentially offer a way to observe indirect billing and improve the accuracy of Medicare payment policies, ranging from quality measurement to ACO attribution, fraud detection algorithms, and productivity measurement. However, eliminating indirect billing of APC-provided care would be the more expedient and accurate way to accurately observe who provides what care to Medicare beneficiaries. For this reason, the Medicare Payment Advisory Commission recommended that Congress require APCs bill Medicare directly.^3^

### Limitations

This study had several limitations. First, our estimates of spending on directly and indirectly billed APC-provided encounters was limited to FFS Medicare. This was due to the absence of payment amounts in the MA encounter data. Second, MA encounter claims are more frequently missing billing NPI, resulting in exclusion from our sample, as we could not ascertain whether the billing provider was a physician or APC. Third,^10,11^ we could not directly link a prescription to a given visit. Rather, we assumed that a prescription filled within 1 day of a visit was associated with that visit. There is misclassification of indirectly billed visits if APCs write prescriptions on behalf of the physicians who provided care (or vice versa). Fourth, we restricted our analysis to a subgroup of APCs (NPs, PA, and CNS) when other types of clinicians (eg, nurse anesthetists, pharmacists, and physical therapists) may also engage in indirect billing. Fifth, our approach relied on prescription drug claims correctly identifying the clinician who prescribed a medication. This may be less true in the few states that severely restrict the prescriptive authority of APCs. As such, we will underestimate the true prevalence of indirect billing within Medicare.

## Conclusions

The results of this cohort study suggest that the amount of indirectly billed encounters provided by APCs is increasing over time and represents a substantial fraction of all care provided by APCs. As APCs represent a growing share of the clinical workforce, identifying APC-provided care that is billed indirectly is integral to understanding who serves Medicare beneficiaries.

## Notes

Supplement 1. eTable 1. Common Services Billed Directly by APPs in 2022 eTable 2. Regression Results Predicting Indirect Billing, 2022 eTable 3. Volume and Spending on APP-Provided Encounters by Billing Status, 2016-2022 eTable 4. Top 12 Most Commonly Indirectly-Billed Services in 2022 eTable 5. Volume of APP and Physician Visits by Billing Status and Payer, 2016-2022 eTable 6. Physician-Level Share of Visits Provided by APPs, 2016-2022 eFigure 1. Methodology for Identifying Incident-To Billing in Medicare Claims eFigure 2. Volume of Directly- and Indirectly-Billed APP Visits by Payer, 2014-2022 eFigure 3. Physician-Level Share of Visits Provided by APPs in 2022, Among Physicians with 50+ Encounters eAppendix. Methodology to Identify Indirect Billing in Medicare Claims

Supplement 2. Data sharing statement
