# Supplementary material for: Quantifying Indirect Billing Within the Medicare Physician Fee Schedule
Source: JAMA Health Forum. 2025 Apr 11;6(4):e250433. doi: 10.1001/jamahealthforum.2025.0433 (PMC11992606; doi:10.1001/jamahealthforum.2025.0433)
Supplement: Supplement 1. — eTable 1. Common Services Billed Directly by APPs in 2022 eTable 2. Regression Results Predicting Indirect Billing, 2022 eTable 3. Volume and Spending on APP-Provided Encounters by Billing Status, 2016-2022 eTable 4. Top 12 Most Commonly Indirectly-Billed Services in 2022 eTable 5. Volume of APP and Physician Visits by Billing Status and Payer, 2016-2022 eTable 6. Physician-Level Share of Visits Provided by APPs, 2016-2022 eFigure 1. Methodology for Identifying Incident-To Billing in Medicare Claims eFigure 2. Volume of Directly- and Indirectly-Billed APP Visits by Payer, 2014-2022 eFigure 3. Physician-Level Share of Visits Provided by APPs in 2022, Among Physicians with 50+ Encounters eAppendix. Methodology to Identify Indirect Billing in Medicare Claims [file jamahealthforum-e250433-s001.pdf]

Supplemental Online Content

Mulcahy JF, Patel SY, Mehrotra A, Neprash HT. Quantifying indirect billings within the Medicare physician fee schedule. *JAMA Health Forum*. 2025:6(4):e250433. doi:10.1001/jamahealthforum.2025.0433

eTable 1. Common Services Billed Directly by APPs in 2022

eTable 2. Regression Results Predicting Indirect Billing, 2022

eTable 3. Volume and Spending on APP-Provided Encounters by Billing Status, 2016-2022

eTable 4. Top 12 Most Commonly Indirectly-Billed Services in 2022

eTable 5. Volume of APP and Physician Visits by Billing Status and Payer, 2016-2022

eTable 6. Physician-Level Share of Visits Provided by APPs, 2016-2022

eFigure 1. Methodology for Identifying Incident-To Billing in Medicare Claims

eFigure 2. Volume of Directly- and Indirectly-Billed APP Visits by Payer, 2014-2022

eFigure 3. Physician-Level Share of Visits Provided by APPs in 2022, Among Physicians with 50+ Encounters

eAppendix. Methodology to Identify Indirect Billing in Medicare Claims

This supplemental material has been provided by the authors to give readers additional information about their work.

© 2025 Mulcahy JF et al. *JAMA Health Forum*.

eTable 1. Common Services Billed Directly by APPs in 2022

HCPCS Code	BETOS Category	% of Directly-Billed APP Services	Description
99214	E/M	24.80%	office o/p est mod 30-39 min
99213	E/M	22.53%	office o/p est low 20-29 min
99203	E/M	2.95%	office o/p new low 30-44 min
17000	Procedure	2.89%	destruct premalg lesion
96372	Treatment	2.52%	ther/proph/diag inj sc/im
99212	E/M	2.49%	office o/p est sf 10-19 min
99204	E/M	2.28%	office o/p new mod 45-59 min
G0439	E/M	2.18%	ppps, subseq visit
17003	Procedure	2.11%	destruct premalg les 2-14
20610	Procedure	1.88%	drain/inj joint/bursa w/o us
99215	E/M	1.84%	office o/p est hi 40-54 min
93000	Test	1.68%	electrocardiogram complete
11102	Procedure	1.67%	tangntl bx skin single les
99211	E/M	1.61%	off/op est may x req phy/qhp
17110	Procedure	1.44%	destruct b9 lesion 1-14
G0444	E/M	0.65%	depression screen annual
51798	Test	0.65%	us urine capacity measure
99442	E/M	0.56%	phone e/m phys/qhp 11-20 min
99490	E/M	0.55%	chrnc care mgmt staff 1st 20
99202	E/M	0.50%	office o/p new sf 15-29 min
11103	Procedure	0.49%	tangntl bx skin ea sep/addl
90833	E/M	0.48%	psytx w pt w e/m 30 min
69210	Procedure	0.47%	remove impacted ear wax uni
73562	Imaging	0.43%	x-ray exam of knee 3
93793	E/M	0.42%	anticoag mgmt pt warfarin
73030	Imaging	0.40%	x-ray exam of shoulder

© 2025 Mulcahy JF et al. *JAMA Health Forum*.

71046	Imaging	0.38%	x-ray exam chest 2 views
17004	Procedure	0.38%	destroy premal lesions 15/>
73502	Imaging	0.37%	x-ray exam hip uni 2-3 views
95117	Treatment	0.35%	immunotherapy injections
99443	E/M	0.33%	phone e/m phys/qhp 21-30 min
73564	Imaging	0.32%	x-ray exam knee 4 or more
96127	E/M	0.31%	brief emotional/behav assmt
99205	E/M	0.28%	office o/p new hi 60-74 min
20611	Procedure	0.27%	drain/inj joint/bursa w/us
72100	Imaging	0.27%	x-ray exam l-s spine 2/3 vws
99497	E/M	0.26%	advncd care plan 30 min
99441	E/M	0.26%	phone e/m phys/qhp 5-10 min
G0442	E/M	0.26%	annual alcohol screen 15 min
97110	Treatment	0.25%	therapeutic exercises
G0438	E/M	0.25%	ppps, initial visit
90471	Treatment	0.24%	immunization admin
96365	Treatment	0.23%	ther/proph/diag iv inf init
97112	Treatment	0.23%	neuromuscular reeducation
G0101	E/M	0.22%	ca screen;pelvic/breast exam
95251	Test	0.21%	cont gluc mntr analysis i&r
73630	Imaging	0.19%	x-ray exam of foot
G0283	Procedure	0.19%	elec stim other than wound
73560	Imaging	0.19%	x-ray exam of knee 1 or 2
20553	Procedure	0.18%	inject trigger points 3/>
99457	E/M	0.18%	rem physiol mntr 1st 20 min
99495	E/M	0.18%	trans care mgmt 14 day disch
93005	Test	0.16%	electrocardiogram tracing
73110	Imaging	0.16%	x-ray exam of wrist
99484	E/M	0.15%	care mgmt svc bhvl hlth cond
99454	E/M	0.15%	rem mntr physiol param dev
G0402	E/M	0.15%	initial preventive exam
73610	Imaging	0.15%	x-ray exam of ankle

© 2025 Mulcahy JF et al. *JAMA Health Forum*.

99496	E/M	0.14%	trans care mgmt 7 day disch
11104	Procedure	0.14%	punch bx skin single lesion
69209	Procedure	0.14%	remove impacted ear wax uni
99406	E/M	0.14%	behav chng smoking 3-10 min
G0181	E/M	0.14%	home health care supervision
73130	Imaging	0.14%	x-ray exam of hand
72110	Imaging	0.13%	x-ray exam l-2 spine 4/>vws
77002	Procedure	0.13%	needle localization by xray
20550	Procedure	0.13%	inj tendon sheath/ligament
20552	Procedure	0.12%	inj trigger point 1/2 muscl
G0180	E/M	0.12%	md certification hha patient
76942	Imaging	0.11%	echo guide for biopsy
90836	E/M	0.11%	psytx w pt w e/m 45 min
99439	E/M	0.11%	chrnc care mgmt staf ea addl
96413	Treatment	0.11%	chemo iv infusion 1 hr
95115	Treatment	0.11%	immunotherapy one injection
90837	E/M	0.11%	psytx w pt 60 minutes
11900	Procedure	0.11%	inject skin lesions </w 7
Q0091	E/M	0.10%	obtaining screen pap smear
17262	Procedure	0.10%	destruction of skin lesions

NOTES: Table presents all services representing at least 0.1% of APP direct billing, in descending order of frequency.

© 2025 Mulcahy JF et al. *JAMA Health Forum*.

eTable 2. Regression Results Predicting Indirect Billing, 2022

Variable	Fee-for-Service Coefficient	Fee-for-Service Standard Error	Medicare Advantage Coefficient	Medicare Advantage Standard Error
Physician Specialty				
Primary Care	Ref		Ref	
Medical	0.1003	0.0001	0.0884	0.0002
Surgical	0.1811	0.0002	0.1476	0.0003
OBGYN	0.1147	0.0006	0.0690	0.0007
Psychiatry	0.0123	0.0004	0.0292	0.0005
BETOS Category				
Evaluation & management	-0.3040	0.0004	-0.2623	0.0005
Procedures	-0.0184	0.0003	0.0116	0.0003
Imaging	0.0007	0.0003	-0.0014	0.0003
DME	0.0568	0.0014	0.0531	0.0013
Anesthesia	-0.1091	0.0133	-0.1661	0.0151
Tests	-0.0066	0.0002	0.0057	0.0002
Treatment	0.0162	0.0002	-0.0014	0.0002
Other	-0.0916	0.0010	-0.0996	0.0010
Patient Census Division				
New England	Ref		Ref	
Middle Atlantic	-0.0252	0.0003	0.0237	0.0005
East North Central	-0.0442	0.0003	-0.0326	0.0005
West North Central	-0.0309	0.0004	-0.0257	0.0006
South Atlantic	-0.0037	0.0003	0.0541	0.0005
East South Central	-0.0162	0.0004	0.0017	0.0005
West South Central	-0.0192	0.0003	0.0120	0.0005
Mountain	0.0199	0.0004	0.0436	0.0005
Pacific	-0.0317	0.0003	-0.0006	0.0005
Territories	-0.0658	0.0006	-0.0791	0.0006
Patient Sex				
Male	Ref		Ref	
Female	0.0079	0.0002	0.0201	0.0001
Unknown	0.7548	0.1070	N/A	N/A
Patient Race				
Non-Hispanic white	Ref		Ref	
Black	-0.0115	0.0003	-0.0310	0.0002
Other	-0.0353	0.0007	-0.0444	0.0009
Asian/ Pacific Islander	-0.0514	0.0003	-0.0577	0.0004
Hispanic	0.0053	0.0003	-0.0051	0.0002
American Indian/ Alaska Native	-0.0103	0.0012	-0.0170	0.0015
Unknown	-0.0204	0.0004	-0.0246	0.0006
Patient Age				
Under 65	0.0146	0.0003	0.0152	0.0003

© 2025 Mulcahy JF et al. *JAMA Health Forum*.

65 -69	Ref		Ref	
70 -74	-0.0070	0.0002	-0.0091	0.0002
75 -79	-0.0143	0.0002	-0.0170	0.0002
80 -84	-0.0201	0.0002	-0.0223	0.0003
85+	-0.0251	0.0002	-0.0266	0.0003
Patient Rurality	0.0259	0.0002	0.0409	0.0002
Patient Disability	0.0245	0.0002	0.0144	0.0002
Patient Dual Eligibility	0.0326	0.0002	0.0401	0.0002
Patient Chronic Conditions				
Acute myocardial infarction	0.0106	0.0009	N/A	N/A
Alzheimer's disease	0.0301	0.0008	N/A	N/A
Alzheimer's related disorders	-0.0095	0.0003	N/A	N/A
Atrial fibrillation	0.0164	0.0003	N/A	N/A
Cataract	-0.0043	0.0002	N/A	N/A
Chronic kidney disease	0.0160	0.0003	N/A	N/A
COPD	0.0224	0.0003	N/A	N/A
Heart failure	0.0113	0.0002	N/A	N/A
Diabetes	0.016	0.0002	N/A	N/A
Glaucoma	-0.0126	0.0003	N/A	N/A
Hip fracture	0.0151	0.0009	N/A	N/A
Ischemic heart disease	0.0023	0.0003	N/A	N/A
Depression	0.0432	0.0003	N/A	N/A
Osteoporosis	0.0002	0.0003	N/A	N/A
Rheumatoid arthritis	0.0217	0.0002	N/A	N/A
Stroke	0.0129	0.0004	N/A	N/A
Breast cancer	0.0074	0.0005	N/A	N/A
Colorectal cancer	0.0034	0.0008	N/A	N/A
Prostate cancer	-0.0002	0.0005	N/A	N/A
Lung cancer	0.0085	0.0009	N/A	N/A
Endometrial cancer	0.0039	0.0013	N/A	N/A
Anemia	0.0228	0.0003	N/A	N/A
Asthma	0.0107	0.0004	N/A	N/A
Hyperlipidemia	0.0012	0.0002	N/A	N/A
Prostatic hyperplasia	-0.0162	0.0002	N/A	N/A
Hypertension	0.0056	0.0002	N/A	N/A
Hypothyroidism	0.0007	0.0001	N/A	N/A
Intercept	0.4116	0.0005	0.3789	0.0007
R^2	0.0938		0.0555	
N=	33,264,311		31,348,493	

NOTES: Table presents coefficients from two linear regressions (on in the fee-for-service population and one in the Medicare Advantage population) of an indicator for indirect billing on physician, patient, and visit characteristics. APP is advanced practice provider.

© 2025 Mulcahy JF et al. *JAMA Health Forum*.

eTable 3. Volume and Spending on APP-Provided Encounters by Billing Status, 2016-2022

**APP-Provided Care**	**2016**	**2017**	**2018**	**2019**	**2020**	**2021**	**2022**
Directly-Billed	28,657,879	32,824,620	38,902,433	46,025,650	42,153,741	52,011,338	59,177,150
Indirectly-Billed	23,538,752	25,641,521	27,786,124	31,036,660	27,729,787	32,287,666	37,605,527
Share Indirectly-Billed	45%	44%	42%	40%	40%	38%	39%
			**Office-Based Allowed Charges (FFS-Only)**				
Directly-Billed	$1,378,699,520	$1,584,789,987	$1,780,341,142	$2,045,855,770	$1,790,839,741	$2,526,125,090	$2,634,025,631
Indirectly-Billed	$1,164,138,875	$1,253,056,463	$1,302,060,649	$1,375,940,394	$1,206,954,650	$1,622,717,129	$1,800,106,031
Share Indirectly-Billed	46%	44%	42%	40%	40%	39%	41%
Medicare savings if billed directly	$174,620,831	$187,958,469	$195,309,097	$206,391,059	$181,043,198	$243,407,569	$270,015,905

© 2025 Mulcahy JF et al. *JAMA Health Forum*.

eTable 4. Top 12 Most Commonly Indirectly-Billed Services in 2022

BETOS Category	CPT Code	Description	Fee Schedule Service
E/M	99214	Office o/p est mod 30-39 min	Yes
E/M	99213	Office o/p est low 20-29 min	Yes
Test	93000	Electrocardiogram complete	Yes
E/M	99204	Office o/p new mod 45-59 min	Yes
E/M	99215	Office o/p est hi 40-54 min	Yes
Treatment	96372	Ther/proph/diag inj sc/im	Yes
E/M	99203	Office o/p new low 30-44 min	Yes
E/M	G0439	Ppps, subseq visit	Yes
E/M	99490	Chrnc care mgmt staff 1st 20	Yes
E/M	99212	Office o/p est sf 10-19 min	Yes
Procedure	20610	Drain/inj joint/bursa w/o us	Yes
Procedure	17000	Destruct premalg lesion	Yes

© 2025 Mulcahy JF et al. *JAMA Health Forum*.

eTable 5. Volume of APP and Physician Visits by Billing Status and Payer, 2016-2022

	2014	2015	2016	2017	2018	2019	2020	2021	20[illegible]
Fee-for-Service									
Prescription-Linked Encounters	46,769,673	48,713,711	50,380,389	50,813,656	51,090,943	49,788,228	40,477,365	41,862,701	41,27[illegible]
APP-Provided and Directly-Billed	4,120,895	5,023,362	5,905,569	6,857,918	7,620,455	8,175,582	6,756,567	7,472,823	8,01[illegible]
Physician-Billed	42,648,778	43,690,349	44,474,820	43,955,738	43,470,488	41,612,646	33,720,798	34,389,878	33,26[illegible]
APP-Provided and Indirectly-Billed	4,297,672	4,689,154	5,026,486	5,367,462	5,565,720	5,629,942	4,797,942	5,212,863	5,39[illegible]
Physician-Provided	38,351,106	39,001,195	39,448,334	38,588,276	37,904,768	35,982,704	28,922,856	29,177,015	27,87[illegible]
Prescription-Unlinked Encounters	180,081,903	177,420,935	178,317,985	176,249,420	173,546,457	173,565,515	146,387,004	159,589,177	157,6[illegible]
APP-Provided and Directly-Billed	14,850,221	15,884,197	18,322,740	20,580,427	22,816,308	25,657,995	23,065,965	27,857,809	29,53[illegible]
Physician-Billed Encounters	166,218,493	161,536,738	159,995,245	155,668,993	150,730,149	147,907,520	123,321,039	131,731,368	128,1[illegible]
APP-Provided and Indirectly-Billed	11,059,730	11,416,221	11,806,769	12,531,634	12,722,122	12,988,188	11,411,204	13,036,136	15,45[illegible]
Physician-Provided	155,158,763	150,120,517	148,188,476	143,137,359	138,008,027	134,919,332	111,909,835	118,695,232	112,7[illegible]
Medicare Advantage									
Prescription-Linked Encounters			17,899,253	19,354,387	25,523,607	30,978,536	28,226,169	33,142,352	38,45[illegible]
APP-Provided and Directly-Billed			1,457,360	1,848,749	2,923,747	4,194,190	4,154,410	5,449,388	7,10[illegible]
Physician-Billed			16,441,893	17,505,638	22,599,860	26,784,346	24,071,759	27,692,964	31,34[illegible]
APP-Provided and Indirectly-Billed			2,344,195	2,727,709	3,604,437	4,480,426	4,178,992	5,042,717	6,01[illegible]
Physician-Provided			14,097,698	14,777,929	18,995,423	22,303,920	19,892,767	22,650,247	25,32[illegible]
Prescription-Unlinked Encounters			38,585,549	40,916,314	52,894,782	64,708,952	59,671,992	72,445,660	84,53[illegible]
APP-Provided and Directly-Billed			2,972,210	3,537,526	5,541,923	7,997,883	8,176,799	11,231,318	14,52[illegible]
Physician-Billed Encounters			35,613,339	37,378,788	47,352,859	56,711,069	51,495,193	61,214,342	70,00[illegible]
APP-Provided and Indirectly-Billed			4,361,302	5,014,716	5,893,845	7,938,104	7,341,649	8,995,950	10,73[illegible]
Physician-Provided			31,252,037	32,364,072	41,459,014	48,772,965	44,153,544	52,218,392	59,26[illegible]
Fee-for-Service + Medicare Advantage									
APP-Provided and Directly-Billed			28,657,879	32,824,620	38,902,433	46,025,650	42,153,741	52,011,338	59,17[illegible]
APP-Provided and Indirectly-Billed			23,538,752	25,641,521	27,786,124	31,036,660	27,729,787	32,287,666	37,60[illegible]

© 2025 Mulcahy JF et al. *JAMA Health Forum*.

Share Indirectly-Billed	45%	44%	42%	40%	40%	38%	3[illegible]
Physician-Billed Visits	256,525,297	254,509,157	264,153,356	273,015,581	232,608,789	255,028,552	262,7[illegible]
Share billed indirectly for APPs	9%	10%	11%	11%	12%	13%	1[illegible]

© 2025 Mulcahy JF et al. *JAMA Health Forum*.

eTable 6. Physician-Level Share of Encounters Provided by APPs, 2016-2022

	2016	2017	2018	2019	2020	2021	2022
10th percentile	0.54%	0.71%	0.90%	1.08%	1.14%	1.43%	1.64%
25th percentile	1.89%	2.20%	2.56%	2.86%	3.03%	3.48%	3.82%
Median	6.45%	7.32%	8.11%	8.97%	9.06%	10.13%	11.11%
75th percentile	18.42%	20.33%	21.60%	23.38%	23.33%	25.56%	27.50%
90th percentile	40.83%	44.00%	44.32%	47.37%	46.88%	50.43%	53.65%

NOTES: This table displays descriptive statistics on the annual distribution of physicians, based on the share of their billed encounters that were provided by APPs.

© 2025 Mulcahy JF et al. *JAMA Health Forum*.

eFigure 1. Methodology for Identifying Incident-To Billing in Medicare Claims

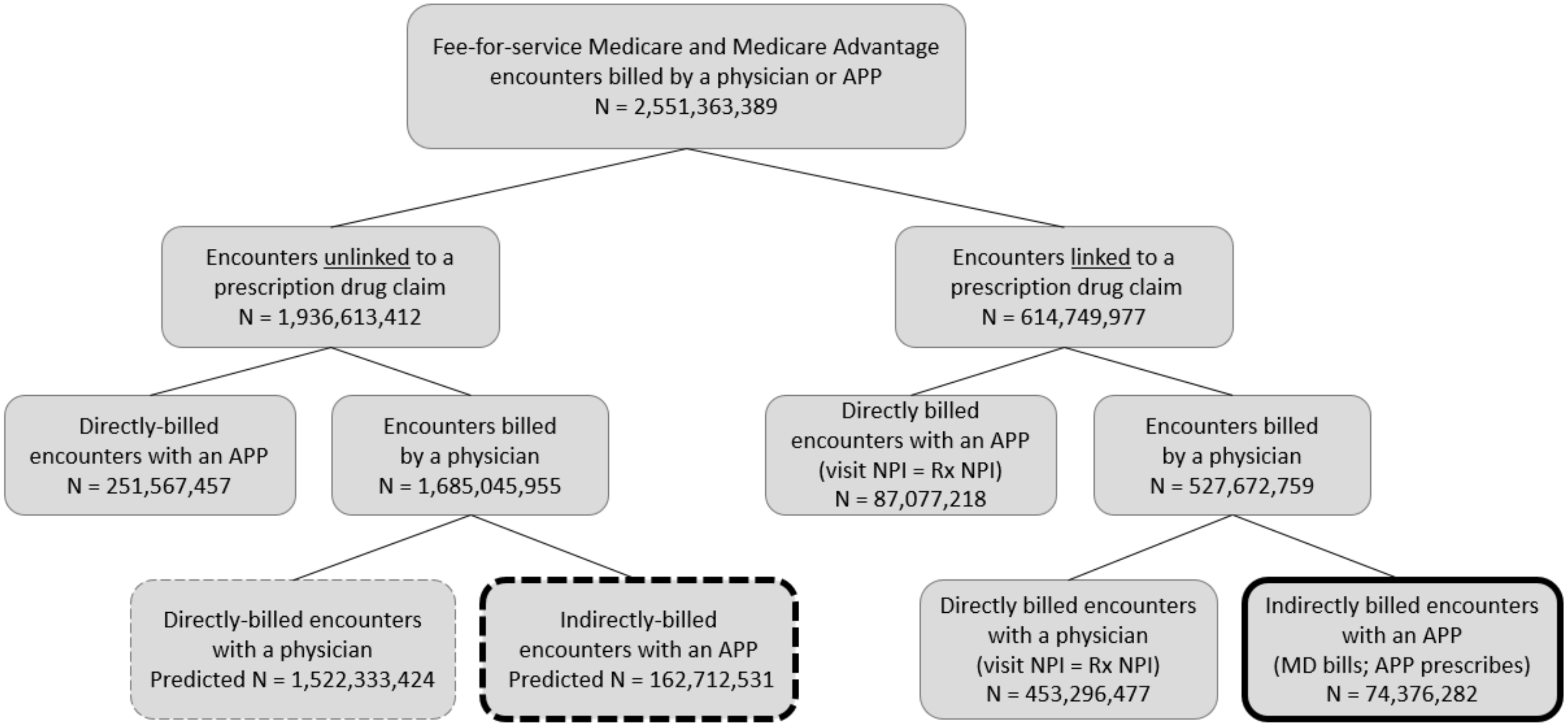

NOTES: APP is advanced practice provider, a category which includes nurse practitioners, physician assistants, and clinical nurse specialists. NPI is National Provider Identifier. Rx is prescription. MD is physician. The solid bolded line identifies indirectly-billed APP-provided encounters identified using the prescription linking method. The bolded dotted line identifies encounters indirectly-billed APP-provided encounters identified by extrapolating patterns of indirect billing to encounters with no associated prescription drug claim.

© 2025 Mulcahy JF et al. *JAMA Health Forum*.

eFigure 2. Volume of Directly- and Indirectly-Billed APP Visits by Payer, 2014-2022

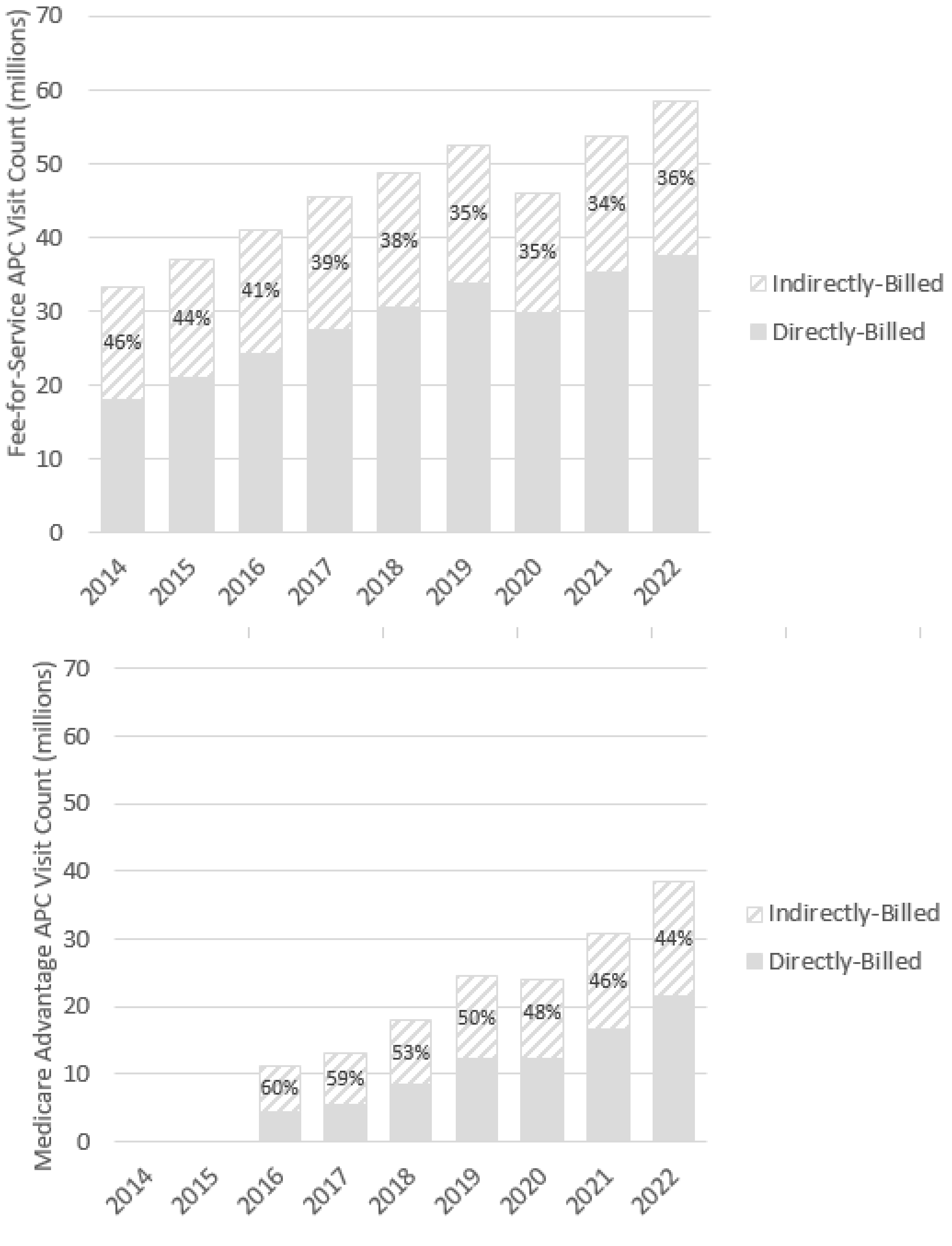

NOTES: This bar graph plots the annual volume and share of all APP-provided visits, by billing status and payer type (i.e., FFS Medicare and Medicare Advantage). APP is advanced practice provider.

© 2025 Mulcahy JF et al. *JAMA Health Forum*.

eFigure 3. Physician-Level Share of Visits Provided by APPs in 2022, Among Physicians with 50+ Medicare Visits

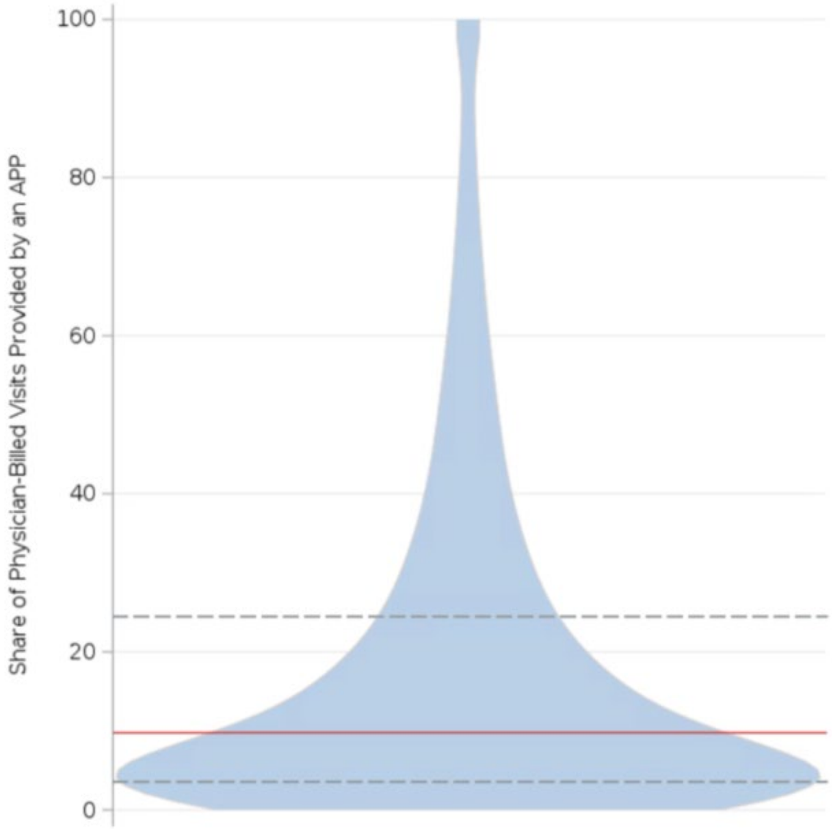

NOTES: This violin plot displays the distribution of physicians by share of prescription-linked visits billed under their NPI and provided by APPs (i.e., billed indirectly), including only physicians with at least 50 Medicare visits in 2022. Calculations include only prescription-linked visits. APP is advanced practice provider.

© 2025 Mulcahy JF et al. *JAMA Health Forum*.

eAppendix. **Methodology to Identify Indirect Billing in Medicare Claims**

This appendix contains seven SAS programs that perform the following functions:

1. Identify office encounters
    a. Fee-for-Service (FFS)
    b. Medicare Advantage (MA)
2. Extract relevant prescriptions
3. Prepare datasets to match encounters to prescriptions
4. Match prescriptions from physicians
5. Match prescriptions from APPs
6. Identify characteristics associated with indirect billing in physician-billed encounters with a linked prescription
7. Predict likelihood of indirect billing in encounters without linked prescriptions

These programs use 100% Medicare claims data to identify indirect billing (i.e., "incident-to" billing under a physician's National Provider Identifier [NPI]) by advanced practice providers (APPs). See below for a narrative description of each SAS program's function and detailed steps. There are separate programs for fee-for-service claims data and Medicare Advantage encounter data. The programs for these two data sources follow the same steps unless otherwise specified.

Program 1A. Create FFS sample of office encounters.

1. *Pull services from carrier file*. For each year the 12 monthly carrier line files are combined into a single dataset. Only lines with a place of service code for "office" (code 11) are kept in the sample. The specialty of the performing provider for each line is categorized has primary care physician, nurse practitioner, physician assistant, clinical nurse specialist, specialty physician, or other based on the line CMS specialty code. The specific specialty code groupings are shown in the following table. Lines that were performed by a provider with a specialty of "other" were excluded from the sample. Lines where the provider NPI was missing were also excluded from the sample.

Specialty Group	CMS Specialty codes
Primary Care Physician	01, 11, 08, 37, 38, 12, 84
Nurse Practitioner	50
Physician Assistant	97
Certified Nurse Specialist	89
Specialty Physician	16, 06, 93, 29, 39, 10, 46, 44, 26, 20, 13, 02, 83, 34, 66, 04, 07, 18, 03, 14, 17, 21, 23, 24, 27,28, 33, 40, 76, 77, 78, 79, 82, 86, 90, 91,98, 99, C0, C03

© 2025 Mulcahy JF et al. *JAMA Health Forum*.

Other	All other codes

2. *Assign BETOS category to every service in sample*. Each carrier line observation has a Healthcare Common Procedure Coding System (HCPCS) code associated with it. These codes are merged with a crosswalk of HCPCS codes to Berenson-Eggers Type of Service (BETOS) categories. This merge allows us to classify each service in to one of eight categories: treatment, test, anesthesia, durable medical equipment, evaluation and management, imaging, procedure, or other.

3. *Identify Physician Fee Schedule services commonly performed by APPs.* Services are merged with a list of Medicare physician fee schedule (PFS) services from the year of service date. A subset of PFS services performed by APPs was then used to generate a list of PFS services that made up ≥ 0.1% of all PFS services performed by APPs.

4. *Group services into encounters*. The carrier line file has separate observations for each service associated with a claim. We group these services into individual encounters using unique combinations of beneficiary ID, encounters date (i.e., claim through date), and performing provider NPI. This step also creates encounter level BETOS and PFS flags that allow the encounter to be associated with multiple BETOS categories based on the type(s) of service(s) performed during that encounter. Encounters can be categorized into multiple BETOS categories, if they contained multiple services. Encounters that did not have any PFS services commonly performed by APPs were dropped from the sample.

Program 1B. Create MA sample of office encounters. Medicare advantage encounter data is structured differently than fee-for-service data, and has some data completeness issues that are not present in FFS data. These differences require that Medicare advantage carrier data be processed differently than FFS data.

1. *Flag beneficiary level enrollment in Medicare advantage contracts with complete data*. MA encounter data relies on plans submitting information on their enrollees' utilization to CMS. Not all plans report this information in a consistent or complete fashion. Jung, Carlin, & colleagues have developed list of MA contracts that report reasonably complete data by comparing MA encounter data to Healthcare Effectiveness Data and Information System (HEDIS) utilization data. The Master Beneficiary Summary File contains information on which MA contract a beneficiary was enrolled in for each month of the year. The list of complete contracts from Jung et al. is used with these MBSF contract enrollment variables to create monthly indicators of whether a beneficiary was enrolled in a contract with complete data or not.

2. *Pull services from carrier file*. Information on the performing provider and the place of service are spread across both the claim level and line level carrier files in MA encounter data. This requires that these two files be merged by the beneficiary ID and the "Encounter Join Key" variables to get all relevant information. There are line level (prvdr_npi) and claim level (rndrng_physn_npi) NPI variables, as well as line level (line_place_of_srvc_cd) and claim level (line_place_of_srvc_cd) place of service variables. For both variables, I check to see if the line level code is populated first. If the line level code is not populated for a procedure, I populate it

with the claim level code. Lines with a place of service other than office (11) or with a missing NPI are excluded from the sample.

3. *Obtain provider specialty data from MD-PPAS*. The MA encounter carrier files do not include information on provider specialty. To obtain provider specialty data the observations from the previous step are linked to the Medicare Data on Provider Practice and Specialty (MD-PPAS) using NPI. The MD-PPAS is a roster of providers who bill Medicare and it contains the same specialty variable found in the FFS carrier files. This specialty variable is used to group provider into primary care physician, nurse practitioner, physician assistant, specialty physician, or other categories. Observations with an "other" specialty category are dropped.

4. *Drop* encounters *from contracts with incomplete data*. Observations are then joined with contract enrollment indicators from step 1 using beneficiary ID. If the enrollment indicator for the month of the observation's claim through date says the beneficiary was not enrolled in a plan with complete data, the observation is dropped from the sample.

5. *Assign BETOS category to every procedure in sample (same as step 2 in program 1A).* Each carrier line observation has a Healthcare Common Procedure Coding System (HCPCS) code associated with it. These codes are merged with a crosswalk of HCPCS codes to Berenson-Eggers Type of Service (BETOS) categories. This merge allows us to classify each procedure in to one of eight categories: treatment, test, anesthesia, durable medical equipment, evaluation and management, imaging, procedure, or other.

6. *Identify Physician Fee Schedule services commonly performed by APPs.* The list of PFS services commonly performed by FFS APPs was merged with our sample of MA services to flag PFS service commonly performed by APPs.

7. *Group procedures into encounters (Same as step 3 in program 1.A).* The carrier line file has separate observations for each procedure associated with a claim. We group these procedures into individual encounters using unique combinations of beneficiary ID, claim through date, and performing provider NPI. This step also creates encounter level BETOS and PFS flags that allow the encounter to be associated with multiple BETOS categories based on the types of procedures performed during that encounter. Encounters that did not have any PFS services commonly performed by APPs were dropped from the sample.

Program 2: Extract relevant prescriptions

1. *Pull prescriptions from relevant prescriber specialties*. Prescription fills are pulled from the part D event file, so they can be linked to encounters. First, the 12 monthly event files from each year are combined into a single dataset. The part D event files do not contain information on prescriber credentials/ specialty, so the NPIs from the event file are linked to the part D

© 2025 Mulcahy JF et al. *JAMA Health Forum*.

prescriber characteristics file. This file is a roster of all provider who wrote a part D prescription in the associated year. The characteristic file contains National Uniform Claim Committee specialty codes which are used to classify prescribers as physicians, physician assistants, nurse practitioners, or other. The codes used to define prescriber specialty can be found in the following table. Prescription fills from a prescriber with an “other” specialty are dropped from the sample.

Specialty Group	NUCC code
Physician	Any code where the first 3 digits are 202, 204, 207, 208, or 209
Physician Assistant	363A00000X, 363AM0700X, 363AS0400X
Nurse Practitioner	Any code other than the 3 PA codes where the first 3 digits are 363
Certified Nurse Specialist	Any code where the first 3 digits are 364
Other	All other NUCC codes

2. *Deduplicate prescriptions by unique combinations of beneficiary ID, fill date, and prescriber NPI*. This is done to account for encounters where multiple prescriptions may have been written. This analysis concerned what type of provider provided an encounter, and the number of prescriptions or type of prescriptions is not relevant. Having only 1 prescription record per fill prevents duplicate matches to encounters.

Certified Nurse Specialist

Program 3: Prepare datasets to match encounters to prescriptions.

1. *Identify prescriptions written by APPs*. Prescriptions that were written by a physician assistant or nurse practitioner are pulled from the dataset created in program 2. Variables that represent the date for 1 day before and 1 day after the fill date are created to be used in the +/- 1 day match with the encounter date.

2. *List beneficiaries who filled a prescription written by an APP*. Beneficiary IDs from the dataset created in the previous step are used to create a list of beneficiaries who filled a prescription from an APP at any point in the year.

3. *Identify encounters for beneficiaries who received a prescription from an APP*. The list of beneficiary IDs from the previous step is used to subset the encounter dataset created in program 1 to only include encounters with beneficiaries who received a prescription from an APP at some point in the year. Note that this dataset will include encounters with both physician and APP NPIs to capture both direct and potentially indirect APP encounters.

4. *Identify prescriptions written by physicians*. Prescriptions that were written by a physician are pulled from the dataset created in program 2. Variables that represent the date for 1 day before and 1 day after the fill date are created to be used in the +/- 1 day match with the encounter date.

© 2025 Mulcahy JF et al. *JAMA Health Forum*.

5. *List beneficiaries who filled a prescription written by a physician*. Beneficiary IDs from the dataset created in the previous step are used to create a list of beneficiaries who filled a prescription from a physician at any point in the year.

6. *Identify physician encounters for beneficiaries who received a prescription from a physician*. The list of beneficiary IDs from the previous step is used to subset the encounter dataset created in program 1 to only encounters for beneficiaries who received a prescription from a physician at some point in the year. Unlike the dataset created in step 3, this dataset is limited to encounters from an NPI with a physician specialty, because we expect all physician prescriptions to come from physician encounters.

Program 4: Match prescriptions from physicians

1. *Merge physician prescriptions and physician encounters*. Match on exact beneficiary ID, exact NPI, and fuzzy (+/- 1 day) service date.

   Deduplicate by beneficiary ID, encounter NPI, and claim through date. This eliminates multiple records created when one encounter matches to multiple prescription events (e.g., a patient has an encounter on Tuesday and fills a prescription on Tuesday and Wednesday). When there are multiple records, exact date matches are prioritized, followed by matches where the prescription fill follows the encounter by one day.

2. *Deduplicate by beneficiary ID, prescription NPI, and prescription fill date*. This eliminates multiple records created when multiple encounters match to the same prescription (e.g., a patient has an encounter on Tuesday and Wednesday and fills a prescription on Tuesday). Deduplicating by both encounter and prescription characteristics ensures every encounter and prescription in our sample is represented only once.

Program 5: Match prescriptions from APPs

1. *Merge prescriptions from APPs with encounters from physicians and APPs*. Match on exact beneficiary ID and fuzzy (+/- 1 day) service date. Note that encounter and prescription NPIs need not match, in order to allow for potential indirectly-billed encounters.

2. *Identify prescriptions where encounter and prescription NPI match*. These are considered directly-billed encounters provided by APPs.

3. *Subset to prescriptions where encounter and prescription NPI did not match*. These are potential indirectly-billed encounters. Delete prescriptions where NPIs are different, but both NPIs are APPs.

4. *Deduplicate both the direct and potentially indirect datasets (created in steps 2 and 3) by beneficiary ID, encounter NPI, and encounter date.* See step 2 of program 4 for additional detail.

© 2025 Mulcahy JF et al. *JAMA Health Forum*.

5. *Deduplicate both the direct and potentially indirect datasets (created in steps 2 and 3) by beneficiary ID, prescription NPI, and prescription fill date*. See step 3 of program 4 for additional detail.

6. *List distinct combinations of beneficiary IDs and encounter NPIs from the potential indirect billing dataset.*

7. *Subset the physician prescription data set created in program 3 to include encounters for the same combinations of beneficiary IDs and encounter NPIs identified in the potentially indirectly-billed dataset (step 6).*

8. *Merge these physician prescriptions with the potential indirect billing dataset created in step 3*. Keep cases that match on beneficiary ID, encounter day +/- 1 day of prescription fill date, and encounter NPI matching the prescription NPI. This creates a dataset of potentially indirectly-billed encounters where the physician that billed for the encounter also wrote a prescription.

9. *Deduplicate the dataset created in step 8 by beneficiary ID, encounter NPI, and encounter date.*

10. *Delete any cases from the potential indirect billing dataset that appeared in the dataset created in step 8.* Because the billing physician also wrote a prescription for the patient in these cases, we assume the physician actually provided the encounter rather than the APP who wrote the matched prescription.

11. *Recombine the direct and indirect APP datasets and create an indirect billing flag.*

Program 6: Identify characteristics associated with indirect billing in physician-billed encounters with a linked prescription. The process in programs 1 through 5 can only identify indirect billing in encounters with an associated prescription. Programs 6 and 7 detail a regression-based approach to extrapolating indirect billing estimates to encounters without a prescription.

1. *Create a file with all physician-billed encounters*. Extract indirectly-billed encounters from the final dataset from program 5 combine these encounters with final dataset from program 4 (physician encounters).

2. *Join physician characteristics.* Merge the dataset from step 1 with the MD-PPAS (joining on NPI) to identify broad specialty categories for the billing physician (primary care, medical specialty, surgical specialty, psychiatry, obstetrics/gynecology, hospital-based specialty, limited liability physicians, and unknown).

3. *Join patient characteristics.* Merge the dataset with the MBSF by beneficiary ID to obtain patient level characteristics, including census division, Research Triangle Institute race group, disability status, dual eligibility for Medicare and Medicaid, five-year age groups, residence in a rural county, and 27 chronic condition flags. NOTE: the chronic condition flags were created using fee-for-service claims, so they cannot be included in the Medicare Advantage regression.

© 2025 Mulcahy JF et al. *JAMA Health Forum*.

4. *Predict indirect billing as a function of encounter, patient, and physician characteristics*. Run an OLS regression with indirect billing as an outcome (1 = indirect billing, 0 = direct billing). Predictors include all beneficiary characteristics from step 3, broad physician specialty, and 8 BETOS category flags.

Program 7: Predict likelihood of indirect billing in encounters without linked prescriptions

1. *Identify physician-billed encounters without a linked prescription fill.* Take the dataset of all encounters created in program 1 and drop all encounters with a linked prescription. Then limit the dataset to physician-billed encounters.

2. *Join physician characteristics*. Merge the dataset from step 1 with MD-PPAS to obtain the same specialty categories from program 6 step 2.

3. *Join patient characteristics*. Merge the dataset with the MBSF to obtain the same patient characteristics from program 6 step 3.

4. *Apply coefficients and impute indirect billing*. Create a dataset that applies regression coefficients from program 6 to the patient, physician, and encounter characteristics to generate a predicted likelihood of indirect billing value for encounters without a linked prescription. Average overall all encounters to generate an implied indirect billing rate.

© 2025 Mulcahy JF et al. *JAMA Health Forum*.
