# Supplementary material for: Quantifying Indirect Billing Within the Medicare Physician Fee Schedule
Source: JAMA Health Forum. 2025 Apr 11;6(4):e250433. doi: 10.1001/jamahealthforum.2025.0433 (PMC11992606; doi:10.1001/jamahealthforum.2025.0433)
Supplement: Supplement 2. — Data sharing statement [file jamahealthforum-e250433-s002.pdf]

Data Sharing Statement

Mulcahy. Quantifying Indirect Billing Within the Medicare Physician Fee Schedule. *JAMA Health Forum*. Published April 11, 2025. doi:10.1001/jamahealthforum.2025.0433

Data
Data available: No

Additional Information
Explanation for why data not available: Data access is governed by a DUA with CMS.
